# Supplementary material for: A Workflow to Create a High-Quality Protein-Ligand Binding Dataset for Training, Validation, and Prediction Tasks
Source: ArXiv. 2025 Mar 7:arXiv:2411.01223v2. Preprint. [Version 2] (PMC11908357)
Supplement: Supplement 1 [file NIHPP2411.01223v2-supplement-1.pdf]

arXiv:2411.01223v2 [physics.bio-ph] 7 Mar 2025

# Supporting Information: A Workflow to Create a High-Quality Protein-Ligand Binding Dataset for Training, Validation, and Prediction Tasks

Yingze Wang[1,Δ], Kunyang Sun[1,Δ], Jie Li[1], Xingyi Guan[1], Oufan Zhang[1], Dorian Bagni[1], Yang Zhang[4−6], Heather A. Carlson[7], Teresa Head-Gordon*[1−3]

[1]Kenneth S. Pitzer Theory Center and Department of Chemistry, [2]Department of Bioengineering, [3]Department of Chemical and Biomolecular Engineering, University of California, Berkeley, CA, 94720 USA
[4]Department of Computer Science, School of Computing, National University of Singapore, 117417, [5]Cancer Science Institute of Singapore, National University of Singapore, 117599, [6]Department of Biochemistry, Yong Loo Lin School of Medicine, National University of Singapore, 117596, Singapore
[7]Odyssey Therapeutics Inc. 1350 Highland Dr., Ann Arbor, MI, 48108, USA
[Δ]authors contributed equally
corresponding author: thg@berkeley.edu

## 1 Data Usage

The HiQBind dataset can be found in the following Figshare repository:

- https://doi.org/10.6084/m9.figshare.27430305

which contains the following files:

- `hiqbind.tar.gz`: Protein-Ligand complexes structures in HiQBind
- `hiqbind_metadata.csv`: Metadata for HiQBind dataset
- `hiqbind_sm_metadata.csv`: Metadata for subset of HiQBind in which ligands all small molecules
- `hiqbind_poly_metadata.csv`: Metadata for subset of HiQBind in which ligands are polyemers
- `README.md`: Description of the structural dataset and columns in the metadata csv file

The metadata files are also provided in the Github repository:

- https://github.com/THGLab/HiQBind/blob/main/figshare

Unzipping the data tarball with command `tar -xzvf hiqbind.tar.gz` will yield two directories `raw_data_hiq_sm` and `raw_data_hiq_poly` corresponding to to the "small molecule" and "polymer" subset of HiQBind, respectively. In each directory, you will see a file structure like this:

```
-- 1a69/
   |-- 1a69_FMB_A_240/
       |-- 1a69_FMB_A_240_ligand.pdb
       |-- 1a69_FMB_A_240_protein.pdb
       |-- 1a69_FMB_A_240_protein_hetatm.pdb
       |-- 1a69_FMB_A_240_hetatm.pdb
       |-- 1a69_FMB_A_240_ligand_refined.sdf
       |-- 1a69_FMB_A_240_protein_refined.pdb
   |-- 1a4m_FMB_B_240/
   |-- 1a4m_FMB_C_240/
-- 1a85/
```

An overview of the file contents is in Table S1 and here is a description of the naming conventions of the files:

- `1a69`: 4-letter PDB ID
- `FMB`: Name of the ligand. If the ligand is a polymer, it will be format like "ACE-DIP", where "ACE" is the name of the first residue and "DIP" is the name of the last residue.
- `A`: Ligand chain ID.
- `240`: Ligand residue number. If the ligand is a polymer, it will be format like "1-3", where "1" is the residue number of the first residue and "3" is the number of the last residue. Note the residue number may contain insertion code, or be a negative integer or zero.

**Table S1:** Overview of the files in HiQBind.

| File | Description |
|---|---|
| *_ligand_refined.sdf | Refined ligand structures (hydrogen added, correct bond order, better tautomer states/protonataion states) with HiQBind-WF workflow |
| *_protein_refined.pdb | Refined protein structures (hydrogen added, missing atoms/residues added) with HiQBind-WF workflow |
| *_ligand.pdb | Ligand structure extracted from the original PDB (not processed) |
| *_protein.pdb | Protein structure extracted from the original PDB (not processed). A protein is defined as chains within 10 angstrom of the ligand structure. |
| *_hetatm.pdb | Additives' structure extracted from the original PDB (not processed) |
| *_protein_hetatm.pdb | Protein structure with additives (solvents, ions) extracted from the original PDB (not processed). Additives are specified with "HETATM" atoms that are within 4 angstroms of the protein chains. |

A description of all fields in the csv-formatted metatdata file in in Table S2.

**Table S2:** Overview of the HiQBind metadata fields.

| Field | Type | Description |
|---|---|---|
| PDBID | string | Four-letter PDB code |
| Resolution | string / float | Resolution of the structure or "NMR" |
| Year | int | Initial release year in RCSB PDB database |
| Ligand Name | string | Name of the ligand.[a] |
| Ligand Chain | string | Chain ID of the ligand. |
| Ligand Residue Number | string | Residue number of the ligand.[b] |
| Binding Affinity Measurement | string | Type of binding affinity assay: "kd", "ki", "ic50" or "ec50".[c] |
| Binding Affinity Sign | string | Sign of the binding affinity measurement: "=", ">=", "<=" or "∼". |
| Binding Affinity Value | float | Value of the binding affinity |
| Binding Affinity Unit | string | Unit of the binding affinity: "fM", "pM", "nM", "uM", "mM" and "M". |
| Log Binding Affinity | float | Binding affinity in log unit. |
| Binding Affinity Source | string | Source of the binding affinity annotations: "BindingMOAD", "BindingDB" or "BioLiP" |
| Binding Affinity Annotation | string | The annotation in the original source. |
| Protein UniProtID | string | UniProtID of the proteins, seperated by a comma if the ligand bound to more than one chain. |
| Protein UniProtName | string | Name of the proteins, separated by a comma if the ligand bound to more than one chain. |
| Ligand SMILES | string | SMILES of the ligand. |
| Ligand MW | float | Molecular weight of the ligand. |
| Ligand LogP | float | LogP value of the ligand computed by RDKit. |
| Ligand TPSA | float | TPSA value of the ligand computed by RDKit. |
| Ligand NumRotBond | int | Number of rotatable bonds in the ligand. |
| Ligand NumHeavyAtoms | int | Number of heavy atoms in the ligand. |
| Ligand NumHDon | int | Number of hydrogen bond donors in the ligand. |
| Ligand NumHAcc | int | Number of hydrogen bond acceptors in the ligand. |
| Ligand QED | float | QED value of the ligand computed by RDKit. |

[a] If the ligand is a polymer, its name will be format like "ACE-DIP", where "ACE" is the name of the first residue and "DIP" is the name of the last residue.

[b] If the ligand is a polymer, its residue number will be format like "1-3", where "1" is the number of the first residue and "3" is the number of the last one. Note the residue number may contain insertion code, or be a negative integer or zero.

[c] In some sources where binding data is labeled as $K_a$ or $K_b$, they are converted to $K_d$ using $K_a = 1/K_d$.

Since users may want to create their own data splits based on time or create subset that does not overlap with exisiting dataset, such as PDBbind v2020, here we provide some relevant analysis in Figure S1. All entries in PDBbind v2020 are before 1/1/2020, and HiQBind contains 1,463 PDB entries deposited after this date. Overall, 11,615 PDB entries out of HiQBind's total 18,160 entries are in PDBbind v2020. This overlapping is due to the

fact that many entries in also appear in Binding MOAD and BindingDB, which are datasets that HiQBind queries binding-affiniy annotation from.

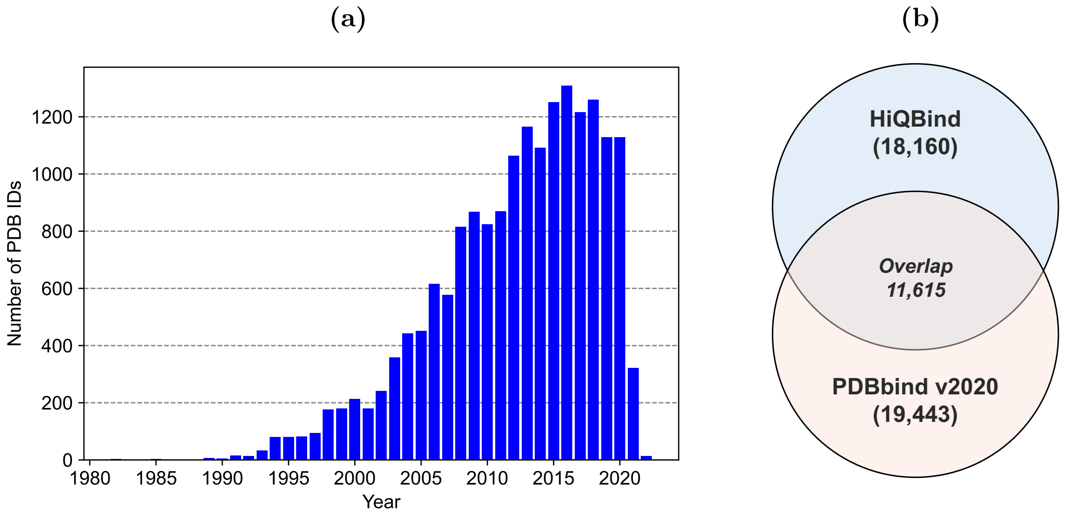


**Figure S1: (a)** The year distribution of PDB entries in HiQBind. HiQBind contains 1,463 PDB entries deposited after 1/1/2020. **(b)** HiQBind PDB entries overlapping with those in PDBbind v2020. Among 18,160 unique PDB IDs in HiQBind, 11,615 of them appear in PDBbind v2020.

# 2 Failed cases in processing HiQBind

**Table S3:** Overview of the number of entries during filter and structure fixing process of HiQBind.

| | Number of unique PDB IDs | | |
|---|---|---|---|
| | Small Molecule | Polymer | Total |
| Before processing | 19105 | 1250 | 20349[a] |
| Successfully processed[d] | 17725 (31572) | 438 (703) | 18160[b] (32275) |
| Failed | 1380 | 812 | 2189[c] |
| Covalent binders | 262 | 16 | 278 |
| Steric clashes | 105 | 7 | 112 |
| Contain uncommon elements | 355 | 6 | 361 |
| Small ligands | 10 | 0 | 10 |
| Fail to fix structures | 638 | 783 | 1428[c] |

[a] 6 PDB entries are included in both small molecule set and polymer set: 1ga8, 1gwv, 1gx4, 1o7o, 1o9f, 4d4u.
[b] 3 PDB entries are sucessfully processed in both small molecule set and polymer set: 1gwv, 1o7o, 1o9f.
[c] 1ga8 and 4d4u are failed in both small molecule set and polymer set; 1gx4 is successful in small molecule set but failed in polymer set.
[d] In parenthesis is the number of protein-ligand structrues.

**278 entries are identified with covalent binders:**

1au0, 1ayu, 1ayv, 1ayw, 1b0f, 1b4e, 1b6a, 1c3b, 1dru, 1drw, 1erm, 1eta, 1etb, 1fsw, 1fsy, 1ga9, 1gfw, 1h2y, 1hbj, 1i72, 1iau, 1kds, 1kdw, 1ke0, 1ke3, 1lhc, 1lhd, 1ms0, 1mt5, 1my8, 1nl6, 1nlj, 1o2t, 1o41, 1o45, 1o4d, 1o4e, 1o4i, 1o5f, 1o5w, 1pi4, 1pi5, 1q6k, 1qfs, 1qhr, 1qm5, 1re1, 1rhm, 1rhr, 1rhu, 1rwm, 1rww, 1s2y, 1s3b, 1s3e, 1snk, 1sri, 1tu6, 1u9v, 1u9w, 1u9x, 1uks, 1w31, 1wof, 1yt7, 2abe, 2alv, 2amd, 2aux, 2auz, 2bxr, 2bxs, 2c72, 2c73, 2c75, 2c76, 2clx, 2dc6, 2dc8, 2dc9, 2dca, 2dcc, 2dcd, 2fj0, 2fs9, 2fzi, 2g5p, 2g5t, 2g63, 2gsu, 2hpp, 2hpq, 2hwp, 2j5f, 2jai, 2jdh, 2jdk, 2op9, 2qky, 2ql5, 2ql7, 2ql9, 2qlb, 2qlq, 2qq7, 2v6n, 2wn2, 2y4a, 2y55, 2yld, 2zu4, 2zu5, 2zz3, 2zz4, 2zz6, 3afk, 3b0r, 3bls, 3dwq, 3dz5, 3fkv, 3g3d, 3g3m, 3gzn, 3hj0, 3i06, 3i4a, 3ika, 3jwe, 3k7f, 3k83, 3k84, 3kjq, 3kw9, 3kwb, 3kwz, 3lok, 3m3c, 3m3e, 3o1g, 3ovx, 3qug, 3rdh, 3sgu, 3sw6, 3u1i, 3w2t, 3zim, 3zs0, 3zs1, 3zvt, 3zvw, 4an0, 4b16, 4bxn, 4d0l, 4dmx, 4e3n, 4hbp, 4hjs, 4hpc, 4i24, 4ll0, 4lqm, 4lrm, 4lv0, 4oyq, 4rus, 4twy, 4wkt, 4x21, 4yqm, 4ywb, 4z16, 4zzm, 4zzo, 5cyi, 5f2e, 5fct, 5feq, 5gmp, 5i3k, 5j87, 5j9y, 5j9z, 5lf3, 5lf7, 5mxq, 5u4f, 5weu, 5xdk, 5xdl, 5xxd, 5xxg, 5y9t, 5yu9, 5za2, 6alj, 6avi, 6ax1, 6b1e, 6bl1, 6c1i, 6cge, 6cha, 6di9, 6e6e, 6ezp, 6hhf, 6hhi, 6iuo, 6j99, 6jpj, 6jwl, 6jx0, 6jx4, 6jxt, 6k1s, 6lw2, 6lze, 6m0k, 6md1, 6mzw, 6o0e, 6o0f, 6oni, 6p69, 6p7i, 6pdz, 6pnm, 6pnn, 6pno, 6qw7, 6r4v, 6rnu, 6swm, 6t5y, 6tfp, 6tpm, 6v6k, 6v6o, 6v9c, 6vh4, 6vim, 6why, 6wi0, 6wtj, 6wtt, 6wxz, 6xd3, 6xhm, 6xmk, 6xr3, 6y2f, 6yi8, 6yzr, 6zpi, 7b3e, 7bwd, 7c6u, 7c8u, 7cb7, 7cbt, 7d1m, 7dpp, 7jkv, 7jxl, 7jxp, 7jxw, 7k0e, 7k1h, 7lel, 7ltn, 7mlf, 7orf, 7rbz, 7rc0, 7tob

**112 entries are identified to exhibit steric clashes:**

1bb0, 1ca8, 1e55, 1fbp, 1h00, 1qga, 1rdy, 1vyr, 1w6p, 1ykl, 1ykp, 1z8a, 2c01, 2ime, 2j9m, 2jjk, 2r24, 2r9m, 2vj1, 2w68, 2w92, 2wnj, 2xuc, 2zyk, 3a23, 3cwd, 3d04, 3fck, 3kfy, 3lb4, 3mdj, 3n8y, 3pch, 3qce, 3qcf, 3rho, 3rv7, 3uo9, 3w4k, 3wzu, 3zrc, 4bii, 4d0m, 4e26, 4ele, 4fh7, 4fm5, 4g5y, 4gs9, 4he9, 4ic0, 4jlm, 4otj, 4ucf, 4v04, 4xi3, 4yx9, 4zx6, 4zzw, 5afk, 5dx3, 5dxb, 5ewa, 5fb7, 5fry, 5glt, 5glz, 5gm0, 5h5o, 5hjg, 5hk9, 5hn7, 5hn9, 5hyr, 5i3b, 5is0, 5ivt, 5sye, 5syf, 5t6p, 5vn1, 5wbf, 5xg4, 5z75,

6a6k, 6ah4, 6ah5, 6c7g, 6ch5, 6co5, 6cup, 6eum, 6gno, 6jz0, 6kiu, 6kiv, 6kix, 6kiz, 6n92, 6n94, 6n96, 6ncf, 6ohu, 6oo8, 6ppl, 6pww, 6qdf, 6ula, 6whv, 6wv1, 7c7h, 7cr4

**361 entries are identified contain ligand with uncommon elements:**

1cp6, 1cws, 1d3v, 1dd7, 1dzj, 1el7, 1el8, 1esz, 1hq5, 1hyv, 1hyz, 1k2v, 1lvk, 1mu9, 1nop, 1rff, 1rfi, 1rg1, 1rg2, 1rgt, 1rgu, 1rh0, 1suo, 1svk, 1tqv, 1tqw, 1v97, 1w0g, 1wva, 1xuf, 1xuj, 1y3g, 1ztz, 2aeb, 2ato, 2bdm, 2bt3, 2c2s, 2c2t, 2cfd, 2ci0, 2cib, 2d0t, 2dy5, 2fb3, 2fdu, 2fdv, 2fdw, 2fou, 2fov, 2foy, 2gj8, 2h7r, 2ij7, 2jjp, 2jld, 2nz5, 2oi4, 2oro, 2orp, 2p8o, 2pll, 2rar, 2rav, 2rb5, 2rbk, 2v0m, 2v96, 2v97, 2w09, 2w0b, 2wfg, 2wh8, 2whf, 2wpu, 2wuz, 2wv2, 2wx2, 2xfh, 2yak, 2ydm, 3b6h, 3b99, 3ben, 3bwf, 3e6k, 3e6v, 3e9b, 3fxz, 3fy0, 3g5n, 3gph, 3i7z, 3i80, 3i8w, 3ixb, 3ixg, 3jt4, 3jt9, 3jus, 3khm, 3koh, 3ksw, 3lc4, 3mdt, 3mke, 3mmr, 3mnu, 3nxu, 3o0j, 3ozu, 3ozv, 3ozw, 3qoa, 3r9c, 3ruk, 3swz, 3t3q, 3t3r, 3t3s, 3t3z, 3tmz, 3vjs, 3vjt, 3wax, 3way, 3zg2, 3zg3, 4c0c, 4c4r, 4c4s, 4coh, 4d2y, 4d2z, 4d30, 4d31, 4d32, 4d33, 4d34, 4d35, 4d36, 4d37, 4d38, 4d39, 4d3a, 4d3b, 4d75, 4d78, 4d7d, 4dtw, 4dtz, 4du2, 4dub, 4due, 4duf, 4ehr, 4ejh, 4eji, 4enh, 4fdh, 4fil, 4g0c, 4g44, 4g45, 4h4d, 4h4e, 4hpd, 4hww, 4hxq, 4hze, 4i06, 4i4g, 4i60, 4ie2, 4ie3, 4iu0, 4iu4, 4ixu, 4ixv, 4j14, 4jfv, 4jfw, 4jjf, 4jjg, 4k9t, 4k9v, 4k9w, 4k9x, 4mdg, 4mdl, 4mdm, 4nkv, 4ob0, 4pf7, 4pk5, 4pk6, 4q3q, 4q3r, 4q3s, 4u72, 4u74, 4uch, 4uhi, 4uhl, 4uqh, 4uzi, 4v3u, 4v3v, 4v3w, 4v3x, 4v3y, 4v3z, 4wmz, 4xrz, 4xud, 4z0l, 4zdy, 4zdz, 4ze0, 4ze1, 4ze2, 4ze3, 4zee, 4zgx, 5agp, 5brv, 5c8i, 5cg5, 5cg6, 5cjh, 5e58, 5e7v, 5ek2, 5ek3, 5ek4, 5etw, 5fom, 5fqb, 5fqc, 5fsa, 5hs1, 5ibe, 5ibf, 5ibg, 5irq, 5irv, 5jqt, 5jws, 5jwu, 5jww, 5k1l, 5k7k, 5l2r, 5l92, 5l94, 5mon, 5moo, 5tz1, 5u48, 5u4a, 5u4c, 5u4e, 5vc0, 5vce, 5veu, 5wmu, 5wn8, 6a16, 6a17, 6abk, 6bbs, 6bc9, 6bcc, 6c6n, 6c8x, 6ceh, 6chi, 6cir, 6ciz, 6cr2, 6d1l, 6d1m, 6da3, 6da5, 6daa, 6dab, 6dac, 6dag, 6daj, 6e40, 6e41, 6f0a, 6ffk, 6frj, 6gcy, 6h1l, 6h1t, 6h3q, 6hwz, 6hx5, 6i96, 6j8r, 6jn6, 6kof, 6kps, 6kvq, 6kw7, 6m7x, 6ma6, 6ma7, 6msn, 6mso, 6o3i, 6pgx, 6pht, 6pu7, 6q2t, 6q37, 6q39, 6qfu, 6qfv, 6qfw, 6qfx, 6qou, 6rpn, 6rvf, 6rvk, 6rvl, 6rw1, 6sp7, 6t7u, 6t9z, 6u30, 6u7o, 6ung, 6unh, 6uni, 6unk, 6unl, 6v7c, 6v7d, 6v7e, 6v7f, 6vbn, 6wpl, 6wr0, 6wr1, 6xz8, 6xz9, 6yzq, 6yzs, 6yzt, 6yzu, 6yzv, 6yzw, 6yzx, 6zjc, 7e0p, 7e7f, 7m8v, 9nse

**10 entries are identified to contain small ligands:**

1lg6, 1ray, 1ugb, 1ugf, 1zdq, 2ca2, 2hds, 4e5q, 4qef, 6pdv

**1428 entries failed the structure fixing:**

11gs, 1a07, 1a2c, 1a5v, 1ad8, 1agm, 1apv, 1apw, 1aqc, 1at5, 1at6, 1axw, 1b11, 1b40, 1b5q, 1b6j, 1bdq, 1bfn, 1bm2, 1bzh, 1c0p, 1c5o, 1c5p, 1c5z, 1cka, 1ckb, 1ckp, 1clu, 1d6s, 1deh, 1dmb, 1e4w, 1e5j, 1eb1, 1ec9, 1eef, 1epq, 1evh, 1eyn, 1ez9, 1f5k, 1f86, 1fh7, 1fh8, 1fh9, 1fhd, 1fls, 1fm1, 1fwu, 1fwv, 1g3c, 1g42, 1g9r, 1ga8, 1gah, 1gai, 1gbn, 1gni, 1gnm, 1gnn, 1gno, 1gny, 1gse, 1gu3, 1gui, 1gvu, 1gvx, 1gwl, 1gwq, 1gwr, 1gww, 1h24, 1h2t, 1h2u, 1h9g, 1hew, 1hkk, 1hpb, 1hps, 1htb, 1htg, 1hut, 1i30, 1i7c, 1i7m, 1igj, 1is0, 1it6, 1j1a, 1j84, 1j8v, 1jd5, 1jfh, 1jlx, 1jn2, 1ju9, 1jvp, 1k1y, 1k7t, 1k7u, 1kjl, 1kjr, 1kl3, 1kl5, 1l6m, 1l7z, 1lax, 1lek, 1lf9, 1lkk, 1lqe, 1lr8, 1lzb, 1lze, 1lzg, 1m7d, 1m7i, 1md2, 1mf4, 1mfa, 1mfd, 1mfl, 1mhw, 1n92, 1nm5, 1nzy, 1oau, 1obx, 1oby, 1ocq, 1of4, 1ogg, 1oh3, 1oh4, 1ols, 1olu, 1olx, 1oxn, 1oxq, 1p2g, 1ph0, 1phw, 1pig, 1pl0, 1pmh, 1ppi, 1pzi, 1pzk, 1q0y, 1q4k, 1qaw, 1qiw, 1qj6, 1qj7, 1qja, 1qjb, 1qlu, 1qsc, 1ra1, 1ra9, 1rbm, 1rbz, 1rc0, 1rcv, 1rd9, 1rdj, 1rdl, 1rdm, 1rdp, 1rf2, 1rr8, 1rrj, 1rsu, 1rx9, 1s7y, 1sem, 1seu, 1shd, 1skx, 1sld, 1sle, 1slg, 1sln, 1ssq, 1str, 1sts, 1syn, 1t7d, 1t8i, 1tb4, 1tc1, 1tf8, 1tlc, 1tps, 1tsd, 1tw6, 1u5b, 1ua7, 1ugx, 1ugy, 1ukq, 1ukt, 1uld, 1ule, 1ulg, 1umw, 1ur8, 1ur9, 1utc, 1ux7, 1uxa, 1uxb, 1uz8, 1uzn, 1v0k, 1v0l, 1v11, 1v16, 1v1m, 1vwl, 1vwn, 1w1g, 1w2h, 1w3k, 1w3l, 1w3y, 1w8f, 1w8h, 1w9d, 1w9e, 1w9o, 1w9w, 1wdn, 1wdr, 1wgc, 1wu1, 1x08, 1x09, 1x7w, 1x7x, 1x7y, 1x7z, 1x80, 1xff, 1xkx, 1xl1, 1xnk, 1xt3, 1xt8, 1xzx, 1y3n, 1y3p, 1y3y, 1y79, 1y7l, 1yf4, 1yhm, 1yp9, 1yxd, 1zfp, 1zhs, 1zkk, 1zky, 2a3y, 2ak5, 2aoc, 2aod, 2aoh, 2aou, 2ar6, 2arb, 2ay7, 2az8, 2az9, 2azb, 2azc, 2bcd, 2bel, 2bgr, 2bmz, 2c05, 2c7u, 2cb8, 2cdp, 2chn, 2cjw, 2cvq, 2dt3, 2e95, 2eax, 2er9, 2euk, 2eum, 2evl, 2ff4, 2fgu, 2fgv, 2foq, 2fos, 2gfa, 2gfj, 2ggu, 2gh9,

2h13, 2h6k, 2h6n, 2h6q, 2hah, 2hj4, 2hjb, 2hl3, 2ho2, 2i74, 2ifw, 2ii3, 2imf, 2iqd, 2itk, 2j1t, 2j6o, 2jg8, 2jjb, 2mip, 2msb, 2ntb, 2nwl, 2nwx, 2nxd, 2nxl, 2nxm, 2o9v, 2oei, 2ok0, 2oor, 2ooz, 2orv, 2p4t, 2peh, 2q5a, 2q7q, 2q8y, 2qmj, 2qt5, 2qta, 2qtz, 2r0h, 2r1w, 2r1x, 2r1y, 2r23, 2r3y, 2rca, 2ri9, 2rkm, 2rkn, 2tpi, 2uue, 2uvh, 2uvi, 2uvj, 2uyq, 2v7d, 2v83, 2v86, 2v88, 2v8l, 2vco, 2vj0, 2vl1, 2vnf, 2vnk, 2vpe, 2vqz, 2vsl, 2vt5, 2vuz, 2vzl, 2w1u, 2w3o, 2w5k, 2w5l, 2w7y, 2wa8, 2wcg, 2wd3, 2wdb, 2wev, 2wgc, 2whp, 2wk2, 2wly, 2wm0, 2wn3, 2wra, 2wt0, 2wt1, 2wt2, 2wyf, 2wyn, 2x4t, 2xd3, 2xg3, 2xg9, 2xog, 2xoi, 2xom, 2xon, 2xqq, 2xwe, 2y4s, 2y64, 2y6g, 2y6l, 2yfz, 2yg0, 2yjq, 2ylg, 2yln, 2ypp, 2z8d, 2z8e, 2z8f, 2zcr, 2zcs, 2zex, 2zey, 2zga, 2zhf, 2zhk, 2zhl, 2zhp, 2zm3, 2zno, 2zq0, 2zy1, 3a06, 3ach, 3aci, 3al3, 3avf, 3ayc, 3ayd, 3b4y, 3b9a, 3bbt, 3bho, 3bib, 3biv, 3boo, 3bpc, 3bwr, 3bzi, 3c0z, 3c3d, 3c9e, 3cbs, 3d3x, 3d6o, 3d9t, 3db3, 3dcq, 3diw, 3drg, 3dz2, 3dz4, 3dz6, 3e7a, 3ebb, 3er3, 3eys, 3f3a, 3f5j, 3f5k, 3f5o, 3f5p, 3f70, 3f81, 3f9w, 3f9y, 3fi2, 3fn0, 3g0e, 3g0f, 3g3r, 3g42, 3gjf, 3gl6, 3gss, 3gxz, 3h9f, 3hkn, 3hlo, 3hzk, 3hzy, 3i6c, 3iet, 3if7, 3iiq, 3iit, 3iqg, 3iqh, 3iqi, 3iqj, 3iqv, 3isd, 3ivv, 3jvk, 3jyr, 3jzj, 3k00, 3k45, 3k8d, 3ka2, 3kce, 3kmc, 3kyf, 3kyg, 3lbl, 3lex, 3lgs, 3ll2, 3lpl, 3lq2, 3lq4, 3lqi, 3lqj, 3lsj, 3lug, 3luh, 3lw1, 3m3o, 3m3r, 3mbp, 3muk, 3mxc, 3mxy, 3n0i, 3n7y, 3n8m, 3n8w, 3nf3, 3nkx, 3nq6, 3nw3, 3nxr, 3ny3, 3o0w, 3o0x, 3o6m, 3oea, 3oeb, 3oka, 3okp, 3old, 3ole, 3olg, 3oli, 3oy8, 3p36, 3pj1, 3q8d, 3qg6, 3qmo, 3qx3, 3qxv, 3qzs, 3r42, 3rjf, 3rme, 3rss, 3rul, 3run, 3ryb, 3sm1, 3sou, 3sow, 3sp5, 3t8v, 3tcg, 3ti4, 3tib, 3tkz, 3tl0, 3tlh, 3u4s, 3u4w, 3u51, 3ur0, 3uvm, 3uwn, 3v0w, 3vf9, 3voz, 3vp1, 3vp2, 3vtr, 3vyk, 3waw, 3wch, 3wel, 3weo, 3wol, 3wom, 3won, 3wqd, 3wqv, 3zdv, 3zkk, 3zq9, 3zst, 3zvy, 3zw2, 3zyb, 3zyh, 3zyr, 3zyx, 4a23, 4a3x, 4a45, 4a7a, 4a9u, 4aa1, 4ad2, 4ad3, 4agl, 4aif, 4ap0, 4aph, 4apr, 4asl, 4auy, 4av0, 4av5, 4avi, 4avj, 4aze, 4b4q, 4b83, 4b9z, 4bdo, 4bgx, 4bh0, 4bj0, 4bnk, 4bqr, 4btl, 4c02, 4c0r, 4c1t, 4c1u, 4c5w, 4c9w, 4cd4, 4cd5, 4cd6, 4cd8, 4cdr, 4cov, 4cow, 4coy, 4coz, 4cqm, 4cub, 4d3w, 4d44, 4d4c, 4d4d, 4d4u, 4dhl, 4dro, 4drq, 4e6c, 4egi, 4ep2, 4eqj, 4er4, 4exh, 4ezq, 4ezt, 4ezy, 4fch, 4fem, 4fgx, 4fk7, 4fut, 4g0u, 4g0w, 4g5f, 4g68, 4gah, 4gk9, 4gxk, 4gzf, 4gzw, 4h39, 4h3b, 4hcz, 4hgw, 4hoe, 4hp0, 4hpi, 4hva, 4i23, 4iaw, 4iax, 4iea, 4ifi, 4igk, 4ii9, 4ikn, 4iwd, 4j48, 4jaw, 4je8, 4jih, 4jir, 4jof, 4jog, 4joh, 4joj, 4jok, 4jor, 4jvn, 4k3l, 4k3m, 4k6y, 4k75, 4k76, 4kup, 4l6t, 4lbj, 4lbk, 4lbl, 4lbm, 4lbn, 4lbo, 4lbt, 4ljh, 4lk7, 4lkd, 4lke, 4lkf, 4lkk, 4lkm, 4lmb, 4lnp, 4lo6, 4m7j, 4m9h, 4mbp, 4mdn, 4mdr, 4mh5, 4mo4, 4mr3, 4mr5, 4mx5, 4n84, 4ngp, 4ngs, 4ngt, 4nmo, 4nmp, 4nmq, 4nmr, 4nms, 4nmt, 4nmv, 4nrt, 4nxq, 4ny3, 4o6l, 4o6w, 4oee, 4oef, 4oeg, 4ojz, 4onc, 4ou3, 4owl, 4oxy, 4oyr, 4p0a, 4p7r, 4p8v, 4p8x, 4p9v, 4p9z, 4pa0, 4pfw, 4pfy, 4phv, 4pk3, 4ptc, 4q4s, 4qhp, 4ql1, 4qlk, 4qll, 4qme, 4qvb, 4qyn, 4r5e, 4r5i, 4r6o, 4r6p, 4r6q, 4r6r, 4r6t, 4r73, 4ra5, 4rdd, 4rh5, 4rmh, 4rx0, 4rxh, 4tqm, 4tro, 4u7w, 4ua8, 4uac, 4ufb, 4up4, 4utv, 4uyw, 4v27, 4w9f, 4w9n, 4wa2, 4wey, 4wq3, 4x1r, 4x3h, 4x3r, 4x3s, 4x9r, 4x9v, 4x9w, 4xbl, 4xbn, 4xbq, 4xoq, 4xur, 4xx9, 4y32, 4y5i, 4yee, 4yef, 4yk0, 4ykj, 4ykk, 4ylz, 4ym0, 4ym2, 4ynl, 4yvv, 4yw2, 4yw8, 4yyt, 4yz5, 4yzc, 4z0u, 4z1n, 4zfz, 4zh7, 4zhc, 4zhl, 4zhm, 4zo7, 4zs9, 4zwy, 4zze, 5a3o, 5a4w, 5ab0, 5ab9, 5aci, 5acw, 5acx, 5ajb, 5aom, 5apr, 5ayf, 5azg, 5b2d, 5bmm, 5bo9, 5boo, 5bpc, 5bs8, 5bta, 5btd, 5btf, 5btr, 5btv, 5bxp, 5bxr, 5bxs, 5bxt, 5c0m, 5c1m, 5cpm, 5cqj, 5cs2, 5cvd, 5cxi, 5d6y, 5d8u, 5d9j, 5dms, 5dxg, 5e1b, 5e1d, 5e1o, 5e4t, 5e50, 5e70, 5e76, 5e7g, 5e8f, 5eel, 5eie, 5elf, 5elq, 5elz, 5em9, 5ema, 5ey9, 5f08, 5f3c, 5f3e, 5f3g, 5f3i, 5f4n, 5f5k, 5f7v, 5f90, 5fa5, 5fa6, 5fiv, 5fjx, 5fjz, 5flc, 5fpp, 5fra, 5fre, 5fu2, 5fu3, 5fvs, 5g4c, 5g5z, 5g61, 5ggo, 5ggp, 5ghv, 5glu, 5glw, 5gmv, 5gu4, 5gwk, 5gx6, 5gx7, 5gza, 5gzk, 5hct, 5hda, 5heb, 5hed, 5hes, 5hey, 5hf1, 5hfb, 5hfc, 5hff, 5hla, 5hlb, 5hld, 5hlp, 5htb, 5htc, 5hza, 5hzb, 5i2f, 5i75, 5icv, 5ifu, 5igm, 5iok, 5izf, 5j41, 5j5y, 5j8u, 5jf2, 5jf3, 5jf4, 5jf5, 5jf7, 5jf8, 5jiz, 5jq7, 5jvi, 5kcf, 5kcw, 5kd9, 5kle, 5klf, 5knj, 5kox, 5l41, 5l4l, 5l79, 5l7f, 5lb7, 5ldq, 5lkc, 5ls7, 5lub, 5lyr, 5m17, 5m1z, 5m28, 5m5d, 5m77, 5may, 5mb0, 5mb1, 5mk9, 5mka, 5mks, 5mm9, 5mnh, 5mrd, 5mtt, 5mtu, 5mwj, 5mxg, 5mxr, 5n16, 5n99, 5n9c, 5nf7, 5nfa, 5npr, 5nps, 5o22, 5o4z, 5o58, 5o7u, 5o7v, 5o7w, 5oca, 5ofl, 5ofx, 5omw, 5on2, 5on3, 5onh, 5osx, 5osy, 5q0l, 5swb, 5sz2, 5t4j, 5t54, 5t55, 5t5j, 5t5l, 5t5p, 5t78, 5t7i, 5t7t, 5t8r, 5tdb, 5tdc, 5tdd, 5tef, 5tha, 5tlm, 5tln, 5tlo, 5tlu, 5tp0, 5tpc, 5ttf, 5ttg, 5ttw, 5tyi, 5tzo, 5u06, 5u0f, 5u1q, 5udk, 5uf1, 5uf4, 5ufc, 5ur1, 5v61, 5v62,

5vdk, 5vnf, 5vrw, 5vrx, 5vry, 5vrz, 5vs1, 5vs2, 5vs3, 5vv8, 5w3y, 5w7i, 5w7x, 5wei, 5wje, 5x2a, 5xo2, 5xof, 5y5u, 5yba, 5yd3, 5yd4, 5yd5, 5ygf, 5yqw, 5ysd, 5yse, 5ysf, 5yto, 5ytu, 5yx2, 5yy4, 5zbz, 5zrf, 6a56, 6a80, 6a8n, 6apr, 6apu, 6asz, 6at0, 6ayh, 6b2c, 6b3p, 6b67, 6bn0, 6brr, 6c19, 6c5h, 6c5j, 6c5k, 6c9n, 6c9p, 6c9r, 6cb5, 6cbz, 6ccu, 6cd8, 6cdg, 6cff, 6cy7, 6d6t, 6dfr, 6dmf, 6dqn, 6dqs, 6dqv, 6dqz, 6dr0, 6drc, 6e3d, 6ecz, 6eeh, 6ek3, 6eor, 6eq1, 6eqv, 6eqw, 6eqx, 6f4p, 6f4r, 6f4t, 6f57, 6f5u, 6f6i, 6f6n, 6f6s, 6f8n, 6fau, 6fav, 6faw, 6fbw, 6fby, 6ffl, 6fft, 6fhu, 6fi4, 6fi5, 6fiv, 6fjj, 6flg, 6fmn, 6fn9, 6fpu, 6fqr, 6fu1, 6fuv, 6g15, 6g9b, 6g9i, 6gd0, 6gdo, 6gex, 6gtx, 6gty, 6gtz, 6gu0, 6gw1, 6gx6, 6h0h, 6h4o, 6h4p, 6h4r, 6h4s, 6h4u, 6h4v, 6h4w, 6h4x, 6h4y, 6h50, 6h51, 6h52, 6h5w, 6hcu, 6hhp, 6hi4, 6hi6, 6hi8, 6hm0, 6hmt, 6how, 6hpg, 6hqx, 6hro, 6hs4, 6i0l, 6i1u, 6i41, 6i5p, 6i5v, 6i5w, 6i68, 6i7a, 6ial, 6iam, 6idg, 6im4, 6inz, 6jad, 6jao, 6jba, 6jue, 6kgo, 6kgq, 6kxa, 6kxb, 6l1f, 6lcf, 6ld3, 6ld4, 6ld5, 6lf2, 6lfj, 6lj2, 6lk4, 6lra, 6lrd, 6m6p, 6m8p, 6mle, 6mlo, 6n19, 6n93, 6n97, 6nlk, 6nll, 6nln, 6nxz, 6ny0, 6o5i, 6o6n, 6orv, 6p02, 6pa7, 6phx, 6pre, 6prg, 6pwv, 6q9w, 6qdx, 6qpl, 6qsw, 6qsx, 6qux, 6r18, 6r3m, 6r6v, 6r6w, 6rav, 6rd2, 6rfh, 6rk4, 6rt6, 6rt7, 6rti, 6ryo, 6s1i, 6sey, 6sgf, 6slo, 6spw, 6spx, 6sq0, 6swn, 6swo, 6swq, 6swx, 6t1d, 6t7z, 6tg4, 6th7, 6tkm, 6tmp, 6tmq, 6tmz, 6tn0, 6tn2, 6tpk, 6tpx, 6tpy, 6tpz, 6tv4, 6twu, 6twx, 6u4q, 6u4t, 6u5m, 6u67, 6u8p, 6u8v, 6u8w, 6u8x, 6u90, 6u91, 6uc3, 6udc, 6v2e, 6vhz, 6vuf, 6vw7, 6wf5, 6wj7, 6wm1, 6wo2, 6wp5, 6wy7, 6x23, 6x3v, 6x9c, 6x9i, 6x9j, 6xwd, 6y2g, 6y58, 6yb4, 6yqn, 6yqo, 6yqr, 6yqs, 6yt1, 6yt2, 6z4q, 6z4s, 6z84, 6zb0, 6zb1, 6zb2, 6zb3, 6zfm, 6zin, 6zpd, 7a5m, 7at8, 7bvt, 7c67, 7c68, 7c69, 7c6g, 7c6i, 7c6k, 7c6m, 7c6t, 7c6w, 7c70, 7jjc, 7k0l, 7k0q, 7k5e, 7k5f, 7k5g, 7k5h, 7kkp, 7kme, 7lan, 7luf, 7n7x, 7otm, 7oty, 7pw4, 7pw5, 7pw6, 7pw7, 8icj, 8ico, 8icw, 8icx, 8icy, 8kme, 9icd, 9icu

**Note**: 1gx4 failed only in small moecule set.

# 3 Failed cases in processing PDBBind

**Table S4:** Overview of the number of entries during filter and structure fixing process of PDBBind.

| | Number of unique PDB IDs | | |
|---|---|---|---|
| | Small Molecule | Polymer | Total |
| Before processing | 16738 | 2705 | 19443 |
| Successfully processed[a] | 14915 (26626) | 746 (1131) | 15661 (27757) |
| Failed | 1823 | 1959 | 3782 |
| Covalent binders | 857 | 101 | 958 |
| Steric clashes | 128 | 37 | 165 |
| Contain uncommon elements | 197 | 9 | 206 |
| Small ligands | 1 | 0 | 1 |
| Fail to fix structures | 640 | 1812 | 2452[c] |

[a] In parenthesis is the number of protein-ligand structrues.

**958 entries are identified with covalent binders:**

1a09, 1a46, 1a5g, 1a61, 1ad8, 1aht, 1amn, 1au0, 1au2, 1auj, 1avp, 1awf, 1awh, 1ayu, 1ayv, 1b0f, 1b5g, 1bgo, 1bio, 1bjr, 1bmq, 1bwn, 1c3b, 1doj, 1e34, 1e37, 1eas, 1eat, 1ekb, 1ero, 1erq, 1exw, 1f1j, 1f7b, 1f9e, 1fsw, 1fsy, 1ft4, 1g37, 1ga9, 1gbt, 1gfw, 1ggd, 1gzg, 1gzv, 1h0w, 1h1b, 1h8y, 1hbj, 1i72, 1i8j, 1iau, 1iem, 1iew, 1inc, 1jlx, 1k2i, 1kds, 1kdw, 1ke0, 1ke3, 1l6s, 1l6y, 1lhc, 1lhd, 1lhe, 1lhf, 1lhg, 1llb, 1me3, 1me4, 1mem, 1mns, 1mpl, 1ms0, 1ms6, 1mwt, 1mxo, 1my8, 1nc6, 1njt, 1nju, 1nkm, 1nl6, 1nlj, 1nms, 1no9, 1npz, 1nqc, 1ny0, 1nyy, 1o2t, 1o41, 1o43, 1o45, 1o4a, 1o4d, 1o4e, 1o4i, 1o4k, 1o5f,

1ong, 1onh, 1p01, 1p02, 1p03, 1p04, 1p05, 1p06, 1p10, 1pau, 1pi4, 1pi5, 1q6k, 1qcp, 1qfs, 1qhr, 1qj1, 1qj6, 1qj7, 1qtn, 1qwu, 1qx1, 1re1, 1rhj, 1rhk, 1rhm, 1rhq, 1rhr, 1rhu, 1rtl, 1rww, 1rwx, 1rxp, 1snk, 1sre, 1sri, 1tbz, 1tlo, 1tmb, 1tu6, 1tyn, 1u9v, 1u9w, 1u9x, 1ukt, 1uod, 1vgc, 1vsn, 1w10, 1w12, 1w14, 1w31, 1x6u, 1y19, 1yk7, 1ylv, 1yly, 1ym1, 1yms, 1yt7, 1z6f, 1zom, 1zpb, 1zpc, 2a4g, 2a4q, 2ajb, 2ajd, 2ajl, 2alv, 2asu, 2aux, 2auz, 2bdl, 2bz5, 2clx, 2eep, 2f9u, 2f9v, 2fda, 2fj0, 2fm2, 2fs8, 2fs9, 2ftd, 2fxr, 2g5p, 2g5t, 2g63, 2g83, 2gbf, 2gbg, 2gph, 2gsu, 2gvf, 2h5d, 2h5i, 2h5j, 2h65, 2ha0, 2hds, 2hob, 2hwo, 2hwp, 2i03, 2i72, 2jal, 2jbv, 2jdh, 2k0x, 2lpr, 2mlm, 2nqg, 2nqi, 2o7e, 2o7v, 2o9a, 2obo, 2oc0, 2oc1, 2oc7, 2op3, 2op9, 2oz2, 2q3z, 2q80, 2q9m, 2q9n, 2qaf, 2qcn, 2qky, 2ql5, 2ql7, 2ql9, 2qlb, 2qlf, 2qlj, 2qlq, 2qnz, 2qq7, 2qve, 2r4b, 2r6n, 2rcx, 2rjr, 2rjs, 2uzj, 2v6n, 2vgc, 2wap, 2wgi, 2wig, 2wij, 2wik, 2wj1, 2wj2, 2woq, 2wzx, 2wzz, 2xcn, 2xdm, 2xdw, 2xe4, 2xk1, 2xlc, 2xln, 2xni, 2xow, 2xu1, 2xu3, 2xu4, 2xu5, 2xzc, 2y2h, 2y2i, 2y2j, 2y2k, 2y2n, 2y2p, 2y4a, 2y55, 2y59, 2yj2, 2yj8, 2yj9, 2yjb, 2yjc, 2z3z, 2zu3, 2zu4, 2zu5, 2zz6, 3a73, 3b1t, 3b1u, 3b9s, 3bar, 3bh3, 3bjm, 3bls, 3blt, 3blu, 3bm6, 3bm8, 3bwk, 3c9e, 3d4f, 3d62, 3dz5, 3e90, 3ewu, 3ex3, 3ex6, 3eyd, 3fkv, 3fmq, 3fmr, 3fnm, 3g3d, 3g3m, 3gjs, 3gpj, 3gzn, 3h0e, 3hd3, 3hha, 3hj0, 3hwn, 3i06, 3i4a, 3ibc, 3ika, 3iut, 3k7f, 3k83, 3k84, 3kjf, 3kjn, 3kjq, 3kqa, 3kw9, 3kwb, 3kwz, 3lj7, 3lle, 3lok, 3lox, 3lpr, 3lxs, 3m3c, 3m3e, 3mbz, 3mkf, 3mxr, 3mxs, 3n4c, 3n5e, 3ns7, 3nzi, 3o1g, 3o6t, 3o86, 3o87, 3o88, 3of8, 3oj8, 3oli, 3opp, 3opr, 3ovx, 3oyp, 3p8e, 3pa8, 3pcb, 3pcf, 3pch, 3pr0, 3qkv, 3qsd, 3rdh, 3rjm, 3s1y, 3s22, 3s3q, 3s3r, 3sji, 3sjo, 3sn8, 3sna, 3snb, 3snc, 3snd, 3sv6, 3sv7, 3sv8, 3svv, 3sz9, 3szb, 3t9t, 3tdz, 3tjm, 3u1i, 3ufa, 3ur9, 3v4j, 3v4x, 3v6r, 3v6s, 3vb4, 3vb5, 3vb6, 3vb7, 3vgc, 3w2p, 3w2q, 3w2t, 3wnr, 3wns, 3wnt, 3zcz, 3zeb, 3zim, 3zmh, 3zmi, 3zmj, 3zot, 3zs0, 3zs1, 3zvt, 3zvw, 4amx, 4amy, 4amz, 4an0, 4an1, 4axm, 4bcb, 4bcc, 4bs5, 4bsq, 4bxn, 4ccd, 4d8e, 4d8i, 4dcd, 4dkt, 4dmy, 4e3i, 4e3j, 4e3k, 4e3l, 4e3m, 4e3n, 4e3o, 4ede, 4eej, 4efg, 4ehm, 4ejf, 4est, 4exz, 4f49, 4fgt, 4fzc, 4fzg, 4gd6, 4ght, 4gk7, 4gkc, 4gs6, 4hbp, 4hcu, 4hcv, 4hnp, 4hrc, 4hrd, 4i7c, 4i7d, 4i9o, 4i9r, 4i9s, 4imq, 4imz, 4inh, 4inr, 4int, 4inu, 4ivk, 4j5p, 4j70, 4jg6, 4jg7, 4jg8, 4jj7, 4jj8, 4jje, 4jmx, 4jr0, 4kqo, 4kw6, 4l0l, 4len, 4lv1, 4lv2, 4lv3, 4lys, 4m1j, 4m8t, 4mao, 4mbf, 4mnv, 4mvn, 4mz4, 4mzo, 4mzs, 4nk3, 4nnn, 4nnw, 4no1, 4no6, 4no8, 4no9, 4o7d, 4ob2, 4ool, 4oon, 4osf, 4pid, 4piq, 4pis, 4pji, 4pkb, 4pl3, 4pl4, 4pl5, 4pnc, 4q1s, 4q2k, 4qbb, 4qkx, 4qps, 4qq5, 4qqc, 4qvl, 4qvm, 4qvn, 4qvp, 4qvq, 4qvv, 4qvw, 4qvy, 4qw0, 4qw1, 4qw3, 4qw4, 4qw5, 4qw6, 4qw7, 4qwf, 4qwg, 4qwi, 4qwj, 4qwk, 4qwl, 4qwr, 4qws, 4qwu, 4qwx, 4qxj, 4qz0, 4qz1, 4qz2, 4qz3, 4qz4, 4qz5, 4qz6, 4qz7, 4qzw, 4qzx, 4r02, 4r17, 4r18, 4r3b, 4r6v, 4rsp, 4ruu, 4s2i, 4tkn, 4tky, 4twy, 4u0g, 4u0x, 4uuq, 4vgc, 4wbg, 4wks, 4wkt, 4wku, 4wkv, 4wm9, 4wmc, 4wsj, 4wsk, 4wx4, 4wx6, 4wx7, 4wyy, 4wz4, 4wz5, 4x0u, 4x21, 4x68, 4x69, 4x6j, 4xbb, 4xbd, 4xcu, 4xjr, 4xuz, 4yas, 4yec, 4yhf, 4yqm, 4yqu, 4yqv, 4yrs, 4yrt, 4yv8, 4z16, 4zro, 5acb, 5ahj, 5c1x, 5c1y, 5c20, 5c91, 5cls, 5cyi, 5d11, 5d6e, 5d6f, 5d9p, 5dg6, 5dgj, 5dp4, 5dp5, 5dp6, 5dp7, 5dp8, 5dp9, 5dpa, 5e0g, 5e0h, 5e0j, 5e7r, 5eb2, 5ee8, 5eec, 5est, 5f02, 5f90, 5fa7, 5fao, 5fap, 5faq, 5fas, 5fat, 5foo, 5fq9, 5g0q, 5gmp, 5gnk, 5gso, 5gty, 5gwa, 5gwz, 5h6v, 5hg5, 5hg7, 5hg8, 5hg9, 5hl9, 5hlb, 5hld, 5i23, 5i24, 5inh, 5j5d, 5j7p, 5j7s, 5j87, 5j8i, 5j8x, 5j9y, 5j9z, 5jh6, 5jk3, 5kre, 5kyk, 5l6h, 5l6i, 5l6j, 5l6o, 5l6p, 5lc0, 5lcj, 5lck, 5lpr, 5mae, 5maj, 5mjb, 5mqy, 5mxq, 5ne1, 5ne3, 5ngf, 5npb, 5nud, 5nwz, 5om9, 5orl, 5swh, 5sys, 5t66, 5t6f, 5t6g, 5tdi, 5teh, 5tg1, 5tg2, 5tg4, 5tg5, 5tg6, 5tg7, 5tig, 5toz, 5tts, 5ttu, 5ttv, 5tyj, 5tyk, 5tyl, 5tyn, 5tyo, 5typ, 5u4f, 5u4g, 5ug8, 5ug9, 5ugc, 5v4q, 5v88, 5vnd, 5vqe, 5vqv, 5vqx, 5vqy, 5vqz, 5w12, 5w13, 5w14, 5wac, 5wad, 5wae, 5waf, 5wag, 5wdl, 5wej, 5wfj, 5x02, 5x5g, 5x79, 5xhr, 5xyz, 5yof, 5yu9, 5za2, 5zde, 5zdg, 5zwf, 6a87, 6af9, 6afa, 6afc, 6afd, 6afe, 6aff, 6afg, 6afh, 6afi, 6afj, 6afl, 6alz, 6ary, 6ax1, 6b0v, 6b0y, 6b1e, 6b1f, 6b1h, 6b1j, 6b1o, 6b1w, 6b1x, 6b1y, 6b41, 6b95, 6bib, 6bic, 6bid, 6bkx, 6bl1, 6bl2, 6bq0, 6cha, 6cn8, 6cqt, 6cqz, 6czu, 6d3g, 6d8e, 6da4, 6db4, 6dge, 6dud, 6e5b, 6e5g, 6e5s, 6e7m, 6ert, 6euv, 6eyz, 6f34, 6f6r, 6fdq, 6fdu, 6ffn, 6ffs, 6fv1, 6fv2, 6g7f, 6g8m, 6g8n, 6g9f, 6g9s, 6gch, 6gcr, 6gop, 6gxy, 6gzy, 6h0u, 6hgy, 6hhg, 6hhh, 6hhi, 6hhj, 6htc, 6htd, 6htp,

6htr, 6hub, 6huc, 6huq, 6huu, 6huv, 6hv4, 6hv5, 6hv7, 6hva, 6hvr, 6hvs, 6hvt, 6hvu, 6hvv, 6hvw, 6i0x, 6ib0, 6ib2, 6ic5, 6ic6, 6iuo, 6iyv, 6iyw, 6j6m, 6jpj, 6k1s, 6lpr, 6m8w, 6m8y, 6m9c, 6m9d, 6m9f, 6mhb, 6mhc, 6mhd, 6mhm, 6mkq, 6mny, 6mu1, 6mzw, 6n4t, 6n9p, 6n9t, 6nd3, 6nng, 6nnr, 6nvg, 6nvh, 6nvi, 6nvj, 6nvl, 6o8i, 6oim, 6ovz, 6p8x, 6p8y, 6p8z, 6pgo, 6pgp, 6pnm, 6pnn, 6pno, 6q35, 6q5b, 6qft, 6qg4, 6qg7, 6qho, 6qhr, 6qmu, 6qw7, 6qw8, 6qw9, 6qwa, 6qwb, 6r4v, 6rjp, 6rmm, 6rn6, 6rn7, 6rn9, 6rne, 6rni, 6rnu, 6rrm, 6rtn, 6s1s, 6s9w, 6s9x, 6skb, 6skd, 6un1, 6un3, 7gch, 7lpr, 8lpr, 9lpr

**165 entries are identified to exhibit steric clashes:**

1ba8, 1bb0, 1ca8, 1e55, 1gj5, 1gvk, 1h9l, 1ibc, 1jbd, 1jrs, 1nu8, 1oxg, 1pop, 1xxh, 1ykp, 1yyy, 1zzz, 2a4r, 2jjk, 2l75, 2p59, 2pre, 2psx, 2q6f, 2qpj, 2qqs, 2r9m, 2vcb, 2vj1, 2w68, 2w92, 2wnj, 2ww0, 2xuc, 3aav, 3atp, 3b3x, 3bcn, 3d04, 3dkj, 3e0p, 3e16, 3fck, 3fv7, 3fyz, 3fzc, 3gjq, 3gjt, 3gpe, 3gxy, 3kl8, 3lce, 3lpg, 3mo0, 3o0u, 3qce, 3qcf, 3qzq, 3rsb, 3rv7, 3sgv, 3t2c, 3tyq, 3uo9, 3wdc, 3wdd, 3wde, 3wzu, 3zmq, 3zrc, 4a2a, 4e26, 4ele, 4f3h, 4g5y, 4ga3, 4gs9, 4h38, 4h3a, 4he9, 4jlm, 4kiu, 4li5, 4lil, 4loj, 4p00, 4ps0, 4ps1, 4qlq, 4qls, 4qlt, 4qlu, 4qlv, 4v04, 4w5j, 4whs, 4ym4, 4yv2, 4yx9, 4z7f, 4z7q, 4zx6, 5afk, 5dpw, 5dx3, 5ewa, 5f5b, 5fb7, 5g1p, 5gsw, 5h5o, 5hk9, 5hn7, 5hn9, 5htb, 5hvp, 5ivt, 5izk, 5jer, 5n6s, 5ngb, 5npf, 5nqe, 5ouh, 5t6p, 5v5o, 5vfn, 5wbf, 5xg4, 5xw6, 5yjo, 5ykp, 5yr4, 5yr5, 5yr6, 5yun, 5z1d, 6a6k, 6a73, 6b22, 6byz, 6c7g, 6ccx, 6dqb, 6eqs, 6eum, 6fmp, 6hmy, 6ijl, 6ikm, 6jij, 6jki, 6jz0, 6k4r, 6kjf, 6myn, 6n55, 6n92, 6n94, 6n96, 6nt2, 6nzg, 6ohu, 6pk7, 6pvs

**206 entries are identified to contain ligand with uncommon elements:**

1cp6, 1d3v, 1dzj, 1esz, 1hq5, 1hyv, 1hyz, 1k2v, 1lkx, 1lvk, 1nxy, 1nym, 1pq3, 1wva, 1y3g, 2aeb, 2cfd, 2cfg, 2fou, 2fov, 2foy, 2jld, 2p8o, 2pll, 2v0c, 2v96, 2wf5, 2wfg, 2wq4, 2yak, 2ydm, 2z97, 3bwf, 3c7n, 3csl, 3cst, 3e6k, 3e6v, 3e81, 3e9b, 3fxz, 3fy0, 3ixg, 3m1s, 3mg0, 3mke, 3mmr, 3mnu, 3o0j, 3p3h, 3p3j, 3p44, 3p55, 3pup, 3q4c, 3qlb, 3qo9, 3rj7, 3ro0, 3sjt, 3skk, 3sl0, 3sl1, 3vjs, 3vjt, 3w8o, 3wax, 3way, 3wc5, 3whw, 3zp9, 4aw8, 4daw, 4dcx, 4dcy, 4ehr, 4fea, 4fil, 4g0c, 4h4d, 4h4e, 4hmq, 4hww, 4hxq, 4hze, 4i06, 4i60, 4ido, 4ie2, 4ie3, 4iu0, 4iu4, 4ixu, 4ixv, 4jfv, 4jfw, 4jhq, 4jjf, 4jjg, 4k6t, 4kai, 4kb7, 4kbi, 4kii, 4l6q, 4ob0, 4ob1, 4q3q, 4q3r, 4q3s, 4rlp, 4u5t, 4xkc, 4z46, 5agi, 5agj, 5agr, 5ags, 5agt, 5dhf, 5fjw, 5fom, 5fqb, 5fqc, 5fsb, 5hj9, 5hja, 5hki, 5hlm, 5ll7, 5m29, 5m2q, 5m34, 5m3b, 5mgi, 5mnx, 5mny, 5mo2, 5mon, 5moo, 5mos, 5nxx, 5nxy, 5od5, 5tgy, 5u48, 5u4a, 5u4c, 5u4e, 5ujo, 5vrl, 5wys, 5zeq, 6abk, 6ajz, 6b30, 6bbs, 6bc9, 6c8x, 6ceh, 6d1l, 6d1m, 6e4v, 6frj, 6h3q, 6hwz, 6hx5, 6i0k, 6i0p, 6i96, 6i97, 6ibs, 6ibv, 6j8q, 6j8r, 6jn3, 6jn4, 6jn5, 6jn6, 6msn, 6mso, 6naf, 6nti, 6ntj, 6pgx, 6pht, 6q2y, 6q30, 6q37, 6q39, 6q92, 6qaf, 6qfu, 6qfv, 6qfw, 6qfx, 6rmf, 6rsa, 6rvf, 6rvk, 6rvl, 6rw1, 6skc, 6u7o, 6u7p, 6uhu

**1 entry are identified to contain small ligands:**

6eu6

**2452 entries failed the structure fixing:**

11gs, 1a07, 1a0t, 1a2c, 1a37, 1a3e, 1abf, 1abt, 1af2, 1agm, 1apb, 1apv, 1apw, 1aqc, 1at5, 1at6, 1atl, 1aze, 1azx, 1b11, 1b2m, 1b40, 1b6j, 1bap, 1bdl, 1bdq, 1bm2, 1bm6, 1bsk, 1bt6, 1bux, 1bzh, 1c5o, 1c5p, 1c5z, 1cka, 1ckb, 1clu, 1cpi, 1cyn, 1czq, 1d4w, 1d6s, 1d8e, 1dkd, 1dmb, 1dva, 1dxp, 1e03, 1e5j, 1eb1, 1ec9, 1eef, 1ej4, 1eoj, 1eol, 1epq, 1eub, 1evh, 1ez9, 1f47, 1f4y, 1f5k, 1ff1, 1fh7, 1fh8, 1fh9, 1fhd, 1fls, 1fwu, 1fwv, 1g42, 1g6g, 1g9r, 1ga8, 1gag, 1gah, 1gai, 1gmy, 1gni, 1gnj, 1gnm, 1gnn, 1gno, 1gny, 1gu3, 1gui, 1gvu, 1gvx, 1gwm, 1gwq, 1gwr, 1gwv, 1gzc, 1h00, 1h07, 1h24, 1h25, 1h26, 1h27, 1h28, 1h2t, 1h2u, 1h5v, 1h6e, 1hc9, 1hgt, 1hkj, 1hkk, 1hkm, 1hps, 1htg, 1i3z, 1i6v, 1i7c, 1i7m, 1i8h, 1i8i, 1idg, 1igj, 1iht, 1ikt, 1ilq, 1iq1, 1is0, 1it6, 1iwq, 1j19, 1j1a, 1j4q, 1jd5, 1jd6, 1jfh, 1jh1, 1jm4, 1jmq, 1jn2, 1jp5, 1jpl, 1juq, 1jvp, 1k1y, 1k9q, 1kat, 1kc5, 1kcs, 1kjr, 1kl3, 1kl5, 1kna, 1kne, 1l6m, 1lek, 1lf8, 1lf9, 1lkk, 1ll4, 1lqe, 1lt5, 1lxh, 1m7d, 1m7i, 1mf4, 1mfa, 1mfd, 1mhw, 1mpa, 1mv0,

1n3w, 1n4m, 1n5z, 1n7m, 1nde, 1ngw, 1nlo, 1nlp, 1nlt, 1ny2, 1o9k, 1oau, 1obx, 1ocn, 1ocq, 1od8, 1oeb, 1ogg, 1oh4, 1oj5, 1ok7, 1oko, 1ols, 1olu, 1olx, 1om9, 1orw, 1osg, 1osv, 1ov3, 1ow6, 1ow7, 1ow8, 1oxn, 1oxq, 1oy7, 1ozv, 1p28, 1p2g, 1p4u, 1pcg, 1pdq, 1ph0, 1pig, 1pl0, 1pmx, 1ppi, 1pum, 1pxh, 1py1, 1pyw, 1pzi, 1q4k, 1qaw, 1qi0, 1qiw, 1qja, 1qjb, 1qm5, 1qsc, 1r17, 1r2b, 1r6z, 1rdj, 1rdl, 1rdn, 1rgj, 1s9v, 1shd, 1sje, 1sld, 1sle, 1slg, 1sln, 1sm3, 1sps, 1ssq, 1str, 1sts, 1szm, 1t29, 1t2v, 1t37, 1t79, 1t7d, 1t7f, 1t7r, 1tc1, 1tet, 1ths, 1tl9, 1tps, 1ttv, 1tyr, 1u8t, 1uef, 1ugx, 1ugy, 1uh1, 1uj0, 1ujj, 1ujk, 1ukh, 1ule, 1ulg, 1umw, 1upk, 1ur9, 1urc, 1urg, 1utc, 1uti, 1uvu, 1ux7, 1uxa, 1uxb, 1uz8, 1v0k, 1v0l, 1v0m, 1v0n, 1v11, 1v16, 1v1m, 1vj6, 1vr1, 1vwl, 1vwn, 1w1g, 1w2h, 1w3k, 1w3l, 1w70, 1w80, 1wdn, 1wdq, 1wdr, 1ws5, 1wu1, 1x11, 1x8s, 1x9d, 1xb7, 1xff, 1xhm, 1xn2, 1xn3, 1xt3, 1xt8, 1y3a, 1y3n, 1y3p, 1y3y, 1ybg, 1ybo, 1yhm, 1ymx, 1yp9, 1yvh, 1ywi, 1yxd, 1yy6, 1z3t, 1z3v, 1zfp, 1zkk, 1zky, 1zub, 2a25, 2aez, 2aof, 2aoh, 2aoi, 2aoj, 2aou, 2aq9, 2auc, 2ay7, 2az8, 2az9, 2azb, 2azc, 2azm, 2b1q, 2b1r, 2b2v, 2b7f, 2bba, 2bcd, 2bgn, 2bgr, 2bmz, 2br8, 2byp, 2c1n, 2c9t, 2ce9, 2cht, 2ci9, 2cia, 2co0, 2d1x, 2d2v, 2df6, 2dwx, 2e7l, 2e95, 2e98, 2e9a, 2e9c, 2eh8, 2emt, 2er0, 2er9, 2euk, 2eum, 2evl, 2ez5, 2f5t, 2f6j, 2fci, 2fgu, 2fgv, 2flu, 2fr8, 2frd, 2fsa, 2fts, 2fuu, 2fx9, 2fys, 2g6q, 2gfa, 2ggu, 2gh9, 2h13, 2h2d, 2h2e, 2h2g, 2h2h, 2h6k, 2h6q, 2h9m, 2h9n, 2h9p, 2hah, 2hdx, 2hj4, 2hjb, 2hkf, 2hmh, 2hrp, 2ig0, 2igv, 2igw, 2itk, 2iv9, 2ivz, 2j7w, 2j9n, 2jb5, 2jbu, 2jdk, 2jdl, 2jg8, 2jjb, 2jk9, 2jkr, 2jkt, 2jmj, 2jnw, 2jq9, 2jqk, 2k1q, 2k3w, 2kgi, 2knh, 2kup, 2l0i, 2l65, 2l7u, 2l8j, 2lcs, 2lct, 2liq, 2llq, 2lp8, 2m0o, 2m3o, 2mg5, 2mip, 2mkr, 2mov, 2mow, 2mpa, 2mwy, 2n3k, 2n7b, 2nn8, 2nwl, 2nwn, 2nxd, 2nxl, 2nxm, 2o9k, 2o9r, 2o9v, 2odd, 2oei, 2oi9, 2ooz, 2peh, 2pem, 2pl9, 2pmc, 2pnx, 2pv3, 2q7q, 2q7y, 2q8y, 2qbx, 2qic, 2qki, 2qmj, 2qt5, 2qta, 2qtr, 2qv7, 2qwe, 2r02, 2r03, 2r05, 2r0h, 2r0y, 2r1w, 2r1x, 2r1y, 2r23, 2r2b, 2r3c, 2r3y, 2r5b, 2r7g, 2rfy, 2ri9, 2rkm, 2rkn, 2rok, 2rol, 2rvn, 2srt, 2tpi, 2uw0, 2uyq, 2uz6, 2v7d, 2v83, 2v85, 2v86, 2v87, 2v88, 2vl1, 2vnf, 2vpe, 2vpg, 2vr3, 2vsl, 2vwf, 2vxj, 2w0p, 2w0z, 2w10, 2w16, 2w2u, 2w3o, 2w47, 2w6c, 2w6t, 2w6u, 2w73, 2w76, 2w77, 2w78, 2w7y, 2w9r, 2wa8, 2wcg, 2wd3, 2whp, 2wk2, 2wly, 2wlz, 2wm0, 2wp1, 2wyf, 2wyn, 2wzf, 2x2i, 2x3t, 2x4t, 2x4z, 2x52, 2x6w, 2x6x, 2x6y, 2x85, 2xaf, 2xag, 2xah, 2xaj, 2xaq, 2xas, 2xcs, 2xct, 2xg3, 2xg9, 2xhs, 2xl2, 2xl3, 2xn6, 2xn7, 2xog, 2xoi, 2xqq, 2xrw, 2xs0, 2xs8, 2xwd, 2xwe, 2xxn, 2xzq, 2y06, 2y07, 2y1n, 2y36, 2y4m, 2y4s, 2y6s, 2y8i, 2y8o, 2y9g, 2y9q, 2ydt, 2yhw, 2yjq, 2ylc, 2yln, 2ymt, 2ynr, 2yns, 2ypp, 2yq6, 2z5o, 2z5s, 2z5t, 2zcr, 2zcs, 2zg3, 2zga, 2zgm, 2zm3, 2zpk, 2zq0, 2zy1, 2zym, 2zyn, 3afk, 3al3, 3alt, 3ap4, 3ap7, 3ary, 3arz, 3as3, 3ask, 3asl, 3au6, 3avf, 3avg, 3avh, 3ax5, 3aya, 3ayc, 3ayd, 3b3s, 3b95, 3bbb, 3bbt, 3bg8, 3bho, 3bim, 3bpc, 3btr, 3bu6, 3bu8, 3bum, 3bun, 3buo, 3buw, 3bux, 3bzi, 3c0z, 3c1n, 3c6w, 3c94, 3cbs, 3cfs, 3cfv, 3ck7, 3ck8, 3ckb, 3coj, 3cs8, 3d1e, 3d1f, 3d3x, 3d45, 3d6o, 3d9k, 3d9l, 3d9m, 3d9n, 3d9o, 3d9p, 3dab, 3dcq, 3diw, 3dla, 3dnj, 3dow, 3dpo, 3drf, 3drg, 3dri, 3ds1, 3ds9, 3dvp, 3dz2, 3dz4, 3dz6, 3e7a, 3e8u, 3ebb, 3eg6, 3ehn, 3eht, 3emh, 3eqs, 3er3, 3ery, 3eu7, 3evc, 3evd, 3evf, 3eyf, 3eys, 3eyu, 3f3a, 3f5j, 3f5k, 3f5l, 3f5p, 3f69, 3f70, 3f81, 3f9w, 3f9y, 3fbr, 3fdm, 3fdt, 3fi2, 3fn0, 3fqa, 3fuc, 3fv8, 3g0e, 3g0f, 3g2s, 3g2t, 3g2u, 3g2v, 3g2w, 3g3r, 3g42, 3g5v, 3g5y, 3g7l, 3gds, 3ggw, 3ghe, 3gl6, 3gsm, 3gss, 3gv6, 3gxz, 3h52, 3h6z, 3h91, 3h9f, 3hkn, 3hkt, 3hlo, 3hqh, 3hs8, 3hs9, 3hzk, 3hzv, 3hzy, 3i02, 3i5r, 3i6c, 3i8t, 3i90, 3i91, 3iet, 3if7, 3ifl, 3ifo, 3ifp, 3iit, 3iiw, 3iiy, 3ij0, 3ij1, 3ijy, 3ikc, 3iqg, 3iqh, 3iqi, 3iqj, 3iqq, 3iss, 3isw, 3iux, 3ivq, 3ivv, 3iw7, 3jpx, 3juq, 3jvk, 3jyr, 3jzg, 3jzh, 3jzj, 3k00, 3k26, 3k27, 3k48, 3k8d, 3ka2, 3kmc, 3krd, 3ktr, 3kyf, 3kyg, 3kze, 3l3q, 3l3x, 3l3z, 3l6x, 3lbl, 3lgl, 3lgs, 3lk1, 3lnj, 3lnz, 3lpl, 3lq2, 3lq4, 3lqi, 3lqj, 3luo, 3m3o, 3m3r, 3m53, 3m54, 3m55, 3m56, 3m57, 3m58, 3m59, 3m5a, 3mbp, 3me9, 3mea, 3met, 3meu, 3ml4, 3mp1, 3mp6, 3muk, 3mxc, 3mxy, 3n5u, 3nf3, 3nfk, 3nfl, 3nii, 3nij, 3nil, 3nin, 3nkx, 3nsn, 3nti, 3nw3, 3ny3, 3o0e, 3o1d, 3o1e, 3o6l, 3o6m, 3ob0, 3ob1, 3ob2, 3odi, 3odl, 3ogx, 3oka, 3okp, 3old, 3ole, 3olg, 3omc, 3omg, 3oq5, 3oy8, 3oyw, 3p4f, 3pdh, 3pfp, 3pgu, 3pj1, 3pkn, 3plu, 3pma, 3poa, 3pp7, 3pqz, 3psl, 3puj, 3puk, 3pxe, 3q5u, 3q6s, 3q8d, 3qfy, 3qfz, 3qg6, 3ql9, 3qlc, 3qmk, 3qn7, 3qnj, 3qo2, 3qs4, 3qxd, 3qxv,

3qzt, 3qzv, 3r42, 3r93, 3rbq, 3rdv, 3rg2, 3rl7, 3rl8, 3rme, 3rqe, 3rqf, 3rqg, 3rtx, 3rul, 3rum, 3run, 3rv6, 3rv8, 3rz9, 3rzi, 3s7f, 3shb, 3shv, 3sm1, 3so6, 3sou, 3sov, 3sow, 3stj, 3sw9, 3sxu, 3szm, 3t5i, 3t6r, 3t7g, 3t83, 3t8v, 3tcg, 3tdu, 3tf6, 3tf7, 3tg5, 3th0, 3ti4, 3tib, 3tiw, 3tkz, 3tl0, 3tlh, 3tpx, 3tsz, 3twr, 3tws, 3twu, 3twv, 3tww, 3twx, 3tzd, 3u3f, 3u78, 3ual, 3uat, 3ud7, 3ud8, 3ud9, 3uda, 3ued, 3uef, 3ueo, 3ui2, 3uig, 3uih, 3uii, 3uij, 3uik, 3upk, 3ur0, 3uri, 3uvk, 3uvl, 3uvm, 3uvn, 3uvo, 3uvu, 3uvw, 3uvx, 3uw9, 3uwl, 3ux0, 3uxg, 3uyr, 3uzd, 3v2o, 3v30, 3v3b, 3v43, 3v4t, 3v7d, 3va4, 3vf9, 3vfj, 3voz, 3vp1, 3vp2, 3vp3, 3vp4, 3vtr, 3vzg, 3w37, 3waw, 3wcb, 3wch, 3wdz, 3wp0, 3wp1, 3wqv, 3wqw, 3wsy, 3wut, 3wuu, 3wuv, 3zdv, 3zev, 3zha, 3zhf, 3zi8, 3zjt, 3zju, 3zjv, 3zke, 3zkf, 3zlv, 3zmp, 3zmt, 3zmu, 3zmv, 3zmz, 3zn0, 3zn1, 3zq9, 3zqi, 3zst, 3zvy, 3zyb, 3zyh, 3zyr, 4a0j, 4a1w, 4a23, 4a4c, 4a50, 4a7j, 4a9t, 4a9u, 4aa1, 4aa2, 4abi, 4abj, 4ad2, 4ad3, 4agl, 4aif, 4aom, 4ap0, 4aph, 4apr, 4auy, 4av0, 4av5, 4avi, 4avj, 4ay6, 4ayp, 4aze, 4b4n, 4b4q, 4b60, 4b83, 4b8o, 4b8p, 4b8y, 4b9h, 4b9w, 4b9z, 4ba3, 4bea, 4bg6, 4bgx, 4blb, 4bpi, 4bpj, 4btl, 4bv2, 4bxu, 4c0r, 4c16, 4c1t, 4c1u, 4c1w, 4c4n, 4c5w, 4c9w, 4cc2, 4cc7, 4cd4, 4cd5, 4cd6, 4cd8, 4cdr, 4ch2, 4ch8, 4ciz, 4cpq, 4cps, 4cpx, 4csy, 4cy1, 4czs, 4d1d, 4d4d, 4de7, 4dhl, 4djs, 4dma, 4dow, 4dro, 4ds1, 4dx9, 4e35, 4e3b, 4e6c, 4e81, 4e9c, 4e9d, 4edu, 4egi, 4elb, 4elg, 4elh, 4ep2, 4eqf, 4eqj, 4er4, 4erq, 4ery, 4erz, 4es0, 4esg, 4ewr, 4exh, 4ezo, 4ezq, 4ezt, 4ezy, 4f14, 4f20, 4fbx, 4fcm, 4fe9, 4fem, 4fgx, 4fgy, 4fk7, 4fmn, 4fmo, 4fmq, 4fn5, 4ft2, 4fut, 4g0a, 4g5f, 4g68, 4g69, 4gah, 4gao, 4gj8, 4glr, 4glx, 4gne, 4gnf, 4gng, 4gq6, 4gvc, 4gvd, 4gw1, 4gw5, 4gwi, 4gxl, 4gy5, 4gye, 4gzf, 4gzw, 4gzx, 4h36, 4h39, 4h3b, 4h3q, 4hcz, 4hfz, 4hgc, 4hp0, 4hpi, 4hpy, 4hs6, 4hs8, 4htp, 4hva, 4hy9, 4hyb, 4i2z, 4i31, 4i32, 4i33, 4i67, 4i7b, 4iaw, 4iax, 4ib5, 4ifi, 4igk, 4igq, 4ii9, 4ikn, 4ipn, 4is6, 4iur, 4iut, 4iuu, 4iuv, 4iwd, 4j09, 4j24, 4j26, 4j2c, 4j3u, 4j48, 4j4v, 4j73, 4j77, 4j7i, 4j84, 4j8g, 4j8r, 4j8s, 4jc1, 4jck, 4je8, 4jfx, 4jfz, 4jg0, 4jg1, 4jiz, 4jjq, 4jmg, 4jmh, 4jof, 4jog, 4joh, 4joj, 4jok, 4k0o, 4k0u, 4k3l, 4k3m, 4k63, 4k64, 4k66, 4k67, 4k6u, 4k6v, 4k6w, 4k6y, 4k72, 4k75, 4k76, 4kc1, 4kc2, 4kc4, 4kmd, 4kn7, 4kom, 4kon, 4ktu, 4kup, 4kvm, 4kx8, 4l1u, 4l58, 4l6t, 4lbl, 4lbo, 4lg6, 4ljh, 4lk6, 4lk7, 4lkd, 4lke, 4lkf, 4lkg, 4lkh, 4lkk, 4lkm, 4ln2, 4lnf, 4lno, 4lnp, 4lp6, 4lq3, 4lte, 4m1d, 4m7j, 4mbp, 4mdn, 4mdr, 4mg5, 4mo4, 4mr3, 4mr5, 4mrd, 4mx5, 4mz5, 4mz6, 4mzf, 4mzh, 4mzj, 4mzk, 4mzl, 4n3w, 4n6g, 4n7g, 4n7h, 4n7j, 4n7y, 4n84, 4nb3, 4ngn, 4ngp, 4ngq, 4ngs, 4ngt, 4nku, 4nl1, 4nmo, 4nmp, 4nmq, 4nmr, 4nms, 4nmt, 4nmv, 4nrk, 4nrl, 4nrt, 4nuf, 4nw2, 4nxq, 4ny3, 4o0r, 4o36, 4o3t, 4o3u, 4o42, 4o45, 4o4y, 4o62, 4o6w, 4oak, 4odk, 4odl, 4odm, 4odn, 4odp, 4odq, 4oee, 4oef, 4oeg, 4oel, 4oem, 4ofl, 4onf, 4oru, 4orx, 4ory, 4ou3, 4ouj, 4ov5, 4oyk, 4oz1, 4p0a, 4p0b, 4p0n, 4p4s, 4pft, 4pfu, 4pgc, 4phv, 4pl6, 4pli, 4pn1, 4pnw, 4po7, 4pry, 4psx, 4ptc, 4pvo, 4pxf, 4pz5, 4pz8, 4q1e, 4q4s, 4q6f, 4qaa, 4qc1, 4qf7, 4qfl, 4qfn, 4qfo, 4qfp, 4qh7, 4qh8, 4qhp, 4ql1, 4qlk, 4qll, 4qme, 4qq4, 4qqi, 4qsk, 4qxt, 4qy8, 4r1e, 4r3s, 4r6t, 4ra1, 4ra5, 4rh5, 4rhu, 4ris, 4rme, 4rqi, 4rqz, 4rrv, 4rxh, 4rxz, 4tk1, 4tk2, 4tk3, 4tk4, 4tmp, 4tnw, 4tt2, 4tw8, 4twt, 4tzm, 4tzn, 4tzq, 4u0a, 4u0b, 4u0c, 4u0d, 4u2w, 4u68, 4u6x, 4u7t, 4u90, 4ua8, 4uac, 4ud7, 4ue1, 4um9, 4umn, 4utn, 4utr, 4utv, 4utx, 4uu5, 4uu7, 4uu8, 4uua, 4uub, 4uw1, 4ux9, 4uxj, 4v1f, 4v27, 4w4z, 4w50, 4w5a, 4w9f, 4w9n, 4wci, 4wey, 4wht, 4why, 4wj7, 4wko, 4wph, 4wq3, 4wrq, 4wv6, 4wy7, 4wym, 4x0z, 4x13, 4x14, 4x1n, 4x1p, 4x1q, 4x1r, 4x1s, 4x34, 4x3e, 4x3h, 4x3i, 4x3k, 4x3r, 4x3s, 4x6h, 4x6s, 4x8n, 4x8p, 4x9r, 4x9v, 4x9w, 4xc2, 4xek, 4xgz, 4xh2, 4xqu, 4xtp, 4xx9, 4xxh, 4xyn, 4y32, 4y3b, 4y5i, 4yb5, 4yc8, 4ydf, 4ydn, 4yee, 4yef, 4yhp, 4yhz, 4yje, 4yjl, 4yk0, 4ykj, 4ykk, 4ym2, 4ynl, 4yoz, 4ysi, 4yw2, 4yy6, 4yyi, 4yym, 4yyn, 4yyt, 4yz5, 4yzc, 4z0d, 4z0e, 4z0f, 4z0u, 4z1n, 4z2o, 4z2p, 4z68, 4z7i, 4z83, 4z88, 4z89, 4z8m, 4zdu, 4zeb, 4zhl, 4zhm, 4znx, 4zs9, 4zwy, 5a0e, 5a2i, 5a2j, 5a2k, 5a3h, 5a3o, 5ab0, 5ab1, 5ab9, 5abp, 5acw, 5acx, 5ajc, 5ajo, 5ajp, 5aom, 5apr, 5awt, 5awu, 5ayf, 5azg, 5b2d, 5b4w, 5b56, 5b6g, 5bjt, 5bmm, 5btr, 5btv, 5c0m, 5c11, 5c13, 5c1m, 5c6v, 5c7e, 5c7f, 5cbm, 5cfa, 5cil, 5cin, 5cqj, 5cqx, 5cr7, 5cs2, 5csz, 5cvd, 5cw8, 5cxi, 5d0j, 5d1u, 5d2a, 5d6y, 5d7e, 5dah, 5dif, 5dms, 5dtj, 5duw, 5dxb, 5dxe, 5dxg, 5e0l, 5e0m, 5e1b, 5e1d, 5e1o, 5e2v, 5e2w, 5e4w, 5e8f, 5eay, 5eel, 5eeq, 5eie, 5ekg, 5elf,

5elq, 5em9, 5ema, 5emb, 5eoc, 5eok, 5epp, 5esq, 5eta, 5etf, 5etu, 5euk, 5ewz, 5ey8, 5ey9, 5eyz, 5ez0, 5f08, 5f2u, 5f3c, 5f3e, 5f3g, 5f3i, 5f4n, 5f67, 5f88, 5fb0, 5fb1, 5ff6, 5fh6, 5fiv, 5fjx, 5fkj, 5fos, 5fpi, 5fpp, 5fyq, 5g5z, 5g60, 5g61, 5g6u, 5gg4, 5ggo, 5ggp, 5ghv, 5glu, 5gmi, 5gmj, 5gmv, 5gp7, 5gs4, 5gtr, 5gu4, 5gwy, 5gx6, 5gx7, 5h1e, 5h5q, 5h5r, 5h5s, 5hct, 5hda, 5heb, 5hed, 5hes, 5hex, 5hey, 5hf1, 5hfb, 5hfc, 5hff, 5hhx, 5hjb, 5hjc, 5hjd, 5hkh, 5hlp, 5hog, 5hpm, 5htc, 5huw, 5huy, 5hyq, 5hyr, 5i25, 5i2f, 5i2i, 5i8c, 5iaw, 5ick, 5icv, 5icx, 5icy, 5icz, 5id0, 5id1, 5ifu, 5igm, 5igq, 5ijj, 5ijp, 5iok, 5iop, 5ir1, 5itf, 5iv2, 5ivn, 5ivz, 5ix1, 5ixt, 5iy4, 5iyv, 5iz6, 5izf, 5izj, 5j19, 5j31, 5j3v, 5j41, 5j5x, 5j7j, 5j8u, 5j9k, 5jek, 5jeo, 5jf2, 5jf3, 5jf4, 5jf5, 5jf7, 5jf8, 5jin, 5jiy, 5jjm, 5jlz, 5jm4, 5jop, 5jq7, 5jqb, 5jr2, 5jvi, 5jy0, 5k5c, 5k6s, 5kez, 5kgn, 5klr, 5klt, 5knj, 5kqd, 5ksu, 5ksv, 5kzp, 5l0c, 5l0h, 5l3f, 5l3g, 5l7f, 5l7k, 5lax, 5lb7, 5lbq, 5lgp, 5lgq, 5lgr, 5lgs, 5lrk, 5lsh, 5lso, 5lu2, 5lub, 5lvx, 5ly1, 5ly2, 5ly3, 5lyr, 5lzh, 5m17, 5m1z, 5m28, 5m5d, 5m63, 5m77, 5mav, 5may, 5mb1, 5mby, 5mgx, 5mk1, 5mk3, 5mk9, 5mka, 5mks, 5mlo, 5mlw, 5mm9, 5mng, 5mnh, 5mo0, 5moq, 5mrd, 5mtw, 5mwj, 5mxo, 5mxr, 5myk, 5myo, 5myx, 5n16, 5n31, 5n7b, 5n7g, 5n7x, 5n8e, 5n8j, 5n8t, 5n8w, 5n99, 5n9n, 5nfa, 5nin, 5njx, 5nne, 5npr, 5nps, 5nw8, 5nwk, 5nx2, 5nxq, 5o22, 5o45, 5o4y, 5o4z, 5o58, 5o5m, 5ocj, 5ods, 5ofx, 5ogl, 5ok6, 5osy, 5ot3, 5oua, 5ous, 5oxk, 5oxl, 5oxm, 5oxn, 5oy3, 5oyd, 5q0l, 5sve, 5svi, 5svx, 5svy, 5svz, 5swf, 5sz2, 5szb, 5szc, 5t1i, 5t1k, 5t1l, 5t1m, 5t31, 5t52, 5t54, 5t6j, 5t78, 5t7s, 5t8r, 5t90, 5tdb, 5tdr, 5tdw, 5tef, 5teg, 5th2, 5th7, 5tha, 5tkj, 5tkk, 5tln, 5tp0, 5tpb, 5tpc, 5tq1, 5tqs, 5ttf, 5ttg, 5ttw, 5twg, 5twh, 5tyi, 5tzo, 5u06, 5u0f, 5u1q, 5u2j, 5u66, 5u6k, 5ufc, 5uff, 5umz, 5un1, 5unj, 5ur1, 5uw5, 5uwi, 5uwj, 5uwp, 5v1d, 5v1y, 5v2p, 5v2q, 5v3r, 5v4b, 5v6y, 5va9, 5vb9, 5vdk, 5vk0, 5vkm, 5vlh, 5vlk, 5vll, 5vlp, 5vnb, 5vqi, 5vtb, 5vzu, 5vzy, 5w0l, 5w0q, 5w38, 5w4e, 5w5s, 5w5u, 5w6i, 5w6r, 5w6t, 5w6u, 5w7i, 5w7j, 5w7x, 5w94, 5wa1, 5wa4, 5wbk, 5wbl, 5wei, 5wg8, 5wgd, 5wgq, 5wir, 5wkl, 5wkm, 5wle, 5wqd, 5wtt, 5wxf, 5wxg, 5wxh, 5wxo, 5wxp, 5wyr, 5x72, 5xgl, 5xhs, 5xhz, 5xo2, 5xof, 5xs8, 5xup, 5xvw, 5xwr, 5xxf, 5xxk, 5xyf, 5y1u, 5y20, 5y21, 5y53, 5y59, 5y5u, 5y5w, 5y6k, 5y7w, 5y97, 5yba, 5yc1, 5yc2, 5yc3, 5yc4, 5yco, 5ygd, 5ygf, 5yjy, 5ypo, 5ypp, 5ypw, 5yqw, 5yto, 5ytu, 5yv5, 5yvx, 5yy4, 5yy9, 5yyz, 5yzd, 5z89, 5z95, 5zbz, 5zia, 5zjy, 5zjz, 5zk5, 5zk7, 5zk9, 5zml, 5znp, 5znr, 5zoo, 5zop, 5zuj, 6a30, 6a5e, 6a6w, 6a80, 6a8g, 6a8n, 6a9c, 6a9o, 6abp, 6aox, 6apr, 6apu, 6ar2, 6asz, 6at0, 6au5, 6ax4, 6axj, 6axk, 6axp, 6ayh, 6ayn, 6azk, 6azl, 6b27, 6b2c, 6b5m, 6b5o, 6b5r, 6b5t, 6b67, 6bcr, 6bcy, 6bd1, 6bgg, 6bhd, 6bhe, 6bhh, 6bhi, 6bij, 6bil, 6bin, 6bir, 6biv, 6bix, 6biy, 6biz, 6bj2, 6bmi, 6bnt, 6buu, 6bvb, 6bvh, 6bw3, 6bw4, 6byk, 6c4u, 6c5h, 6c5j, 6c5k, 6c9n, 6c9p, 6c9r, 6cb5, 6cct, 6ccu, 6cd8, 6cdg, 6cdm, 6cdo, 6cdp, 6cer, 6cf6, 6cgt, 6cjv, 6co4, 6d07, 6d08, 6d1u, 6d3x, 6d3y, 6d3z, 6d40, 6d4o, 6d6t, 6df1, 6df2, 6do5, 6drt, 6dub, 6e49, 6e5x, 6e8k, 6e8m, 6ecz, 6eeh, 6egw, 6eiz, 6ek3, 6em6, 6em7, 6ema, 6eo0, 6epy, 6epz, 6eq1, 6eqv, 6eqw, 6eqx, 6er3, 6eru, 6esa, 6evp, 6eww, 6ex0, 6ezi, 6f08, 6f09, 6f55, 6f5m, 6f5u, 6f6d, 6f6i, 6f6n, 6f6s, 6f7t, 6f8g, 6fam, 6fau, 6fav, 6faw, 6fbw, 6fby, 6fc6, 6fel, 6fhu, 6fi4, 6fi5, 6fiv, 6fkp, 6fkq, 6fky, 6fkz, 6flg, 6fmn, 6fn9, 6fpu, 6fsd, 6fse, 6fu1, 6fvn, 6fx1, 6fzf, 6fzj, 6fzp, 6g0q, 6g15, 6g2n, 6g47, 6g6x, 6g84, 6g85, 6g86, 6g8i, 6g8j, 6g8k, 6g8l, 6g8p, 6g8q, 6g9b, 6g9i, 6gfx, 6ggb, 6gjj, 6gw1, 6gwe, 6gxe, 6gzl, 6h0b, 6h41, 6h4o, 6h4p, 6h4r, 6h4s, 6h4u, 6h4v, 6h4w, 6h4x, 6h4y, 6h50, 6h51, 6h52, 6h5w, 6h7b, 6h8c, 6h96, 6h9v, 6hck, 6hcu, 6hhp, 6hks, 6hlb, 6hld, 6hle, 6hm4, 6hmg, 6hmt, 6hoi, 6hol, 6hpg, 6hro, 6hs4, 6hv2, 6hy7, 6hza, 6hzb, 6hzc, 6hzd, 6hzx, 6i41, 6i4x, 6i5j, 6i5n, 6i5p, 6i68, 6i7a, 6iae, 6iam, 6idg, 6iiw, 6im4, 6inz, 6iqg, 6iso, 6j9w, 6j9y, 6jad, 6jag, 6jam, 6jan, 6jao, 6jap, 6jax, 6jb0, 6jb4, 6jbb, 6jjm, 6jjn, 6jjz, 6k2n, 6k5r, 6k5t, 6kdi, 6kmj, 6md6, 6me1, 6mil, 6mim, 6min, 6miq, 6mle, 6mlo, 6mm5, 6mnf, 6mqc, 6mqe, 6mqm, 6msy, 6mtv, 6mu3, 6mub, 6n19, 6n3e, 6n3f, 6n5x, 6n7q, 6n87, 6n93, 6nao, 6ncp, 6njz, 6nk0, 6nk1, 6nkp, 6nsx, 6nxz, 6ny0, 6o21, 6o3w, 6o3x, 6o3y, 6o7g, 6oie, 6om2, 6om4, 6oxl, 6p3w, 6p7p, 6p7q, 6pek, 6peu, 6phx, 6pi7, 6pit, 6prg, 6pxc, 6q38, 6q4q, 6q9t, 6q9w, 6qc0, 6qcg, 6qdx, 6qk8, 6qpl, 6qs1, 6qsz, 6qtm, 6qto, 6qtq, 6qtr, 6qts, 6qtw, 6qtx, 6qzr, 6r0x, 6r8i, 6rhe, 6rk4, 6rml, 6rr0, 6s07, 6sen, 6sq0, 6tyz,

6u5m, 6uyx, 6uyy, 6uyz, 6v1c, 7abp, 7kme, 8abp, 9abp, 9icd
